# Seroprevalence of infectious bronchitis virus and avian reovirus in free backyard chickens

**DOI:** 10.4102/ojvr.v89i1.2042

**Published:** 2022-11-11

**Authors:** Sonia C. Pinto, Jescka Aleixo, Kleidy Camela, Abel G. Chilundo, Custódio G. Bila

**Affiliations:** 1 Department of Animal Health and Epidemiology, Faculty of Veterinary Medicine, Eduardo Mondlane University, Maputo, Mozambique

**Keywords:** infectious bronchitis virus, avian reovirus, prevalence, backyard chickens, Mozambique, Africa

## Abstract

Infectious bronchitis virus (IBV) and avian reovirus (ARV) cause significant losses in the poultry industry throughout the world. A cross-sectional study was conducted in four villages in Manjacaze district, Southern Mozambique, to determine the seroprevalence of IBV and ARV. A total of 467 serum samples from adult unvaccinated backyard chickens were screened using commercial and competitive enzyme-linked immunoabsorbent assay kits. Our results showed anti-IBV and anti-ARV antibodies in all surveyed households and villages. The overall seroprevalence was 89.5% (95% confidence interval [CI]: 77.2–97.4) and 95.7% (95% CI: 88.0–99.2) for IBV and ARV, respectively. The risk of becoming exposed to IBV was lower in Chidenguele village compared with the other three villages (*p* > 0.05). However, no statistically significant differences were observed for becoming exposed to ARV between villages (*p* < 0.05). The backyard chickens tested in this study had no previous history of vaccination, outbreaks or typical clinical signs of IB and AR diseases. Therefore, the presence of antibodies to IBV and ARV was considered clear evidence that the birds have been naturally exposed to those two infectious agents, and the infection was of subclinical type. It is concluded that IBV and ARV are widespread in backyard chickens in the studied area. These obtained data are essential for design and implementation of chicken health development programmes.

**Contribution:**

The epidemiology of IBV and ARV of backyard chicken in Mozambique is unknown. This study determined the seroprevalence of IBV and ARV in backyard chicken health. The obtained data are essential for design and implementation of chicken health development programmes.

## Introduction

Infectious bronchitis (IB) is a highly contagious disease with severe economic consequences in the chicken industry worldwide (Cavanagh 2007). The etiologic agent, infectious bronchitis virus (IBV), belongs to the family *Coronaviridae* and subfamily *Coronavirinae* within the genera of *Gammacoronaviridae.* Infectious bronchitis virus is an enveloped virus with single-stranded positive-sense linear ribonucleic acid (RNA) with the genome of about 27 kB (Jackwood 2012). Birds infected with IBV may show depression, watery eyes, mucus in the nares and trachea, gasping, coughing and tracheal rales (Jackwood 2012). In laying birds, IB disease can also cause a decrease in egg production and egg quality (Cavanagh & Naqi 2003; Jackwood, Hall & Handel 2012). Morbidity is normally 100%, while mortality is low but can be higher than 50%, depending on the age of the birds, strain of the virus and presence of secondary infections (Jackwood 2012; Jackwood et al. 2012). At necropsy, gross lesions observed on respiratory organs of chickens naturally infected with IBV include the presence of mucous secretion, congestion and hyperaemia in the trachea and mild focal areas of lung consolidation (Khataby et al. 2016). In backyard chickens, IB has been reported in a few countries worldwide, including Zimbabwe (Kelly et al. 1994), South Africa (Thekisoe et al. 2003), Botswana (Mushi et al. 2006), Nigeria (Owoade, Ducatez & Muller 2006), Ethiopia (Chaka et al. 2012), Ivory Coast (Kouakou et al. 2015), Mexico (Gutierrez-Ruiz et al. 2000), Switzerland (Keller-Berger & Hoop 1993), Bangladesh (Biswas et al. 2009) and Iran (Hadipour et al. 2011). There are no published reports of this disease in chickens in Mozambique.

Avian reovirus (ARV) is a pathogenic agent in chickens (*Gallus gallus*) and a member of the genus *Orthoreovirus* in the Reoviridae family. The ARV genome contains 10 segments of double-stranded RNA that encode at least eight structural proteins (lA, lB, lC, mA, mB, sA, sB and sC) and four nonstructural proteins (mNS, P10, P17 and sNS) (Bodelon et al. 2001; Saif et al. 2003; Wundervald & Hoop 2002). Reovirus infections are prevalent worldwide in chickens, turkeys and other avian species (Bodelon et al. 2001), where they are associated with several diseases such as viral arthritis or malabsorption syndrome, causing considerable economic losses in the chicken industry worldwide (Jackwood 2012; Jackwood et al. 2012; Khataby et al. 2016). Economic losses related to reoviral infections are frequently associated with increased mortality, viral arthritis or tenosynovitis and general lack of performance (Jackwood 2012; Jackwood et al. 2012; Khataby et al. 2016). Literature on the epidemiology and significance of ARV infections in backyard chickens is limited to only one report from Zimbabwe (Kelly et al. 1994). To our knowledge, there are no reports of ARV presence in either backyard, broiler or layer chickens in Mozambique.

Several government and nongovernmental backyard chicken development and social projects are being implemented in Southern Mozambique. The aim of these initiatives is to improve availability of protein of animal origin and income generation for villagers. Data on disease prevalence are needed for design and implementation of chicken disease control strategies.

Unlike other viral and bacterial diseases (Messa et al. 2017; Taunde et al. 2017a, 2017b), little is known about the IB and AR status of backyard chickens in Mozambique. This study aimed to determine the seroprevalence of IBV and ARV in backyard chicken health in Southern Mozambique.

## Research methods and design

### Study area and sampling

**FIGURE 1:**
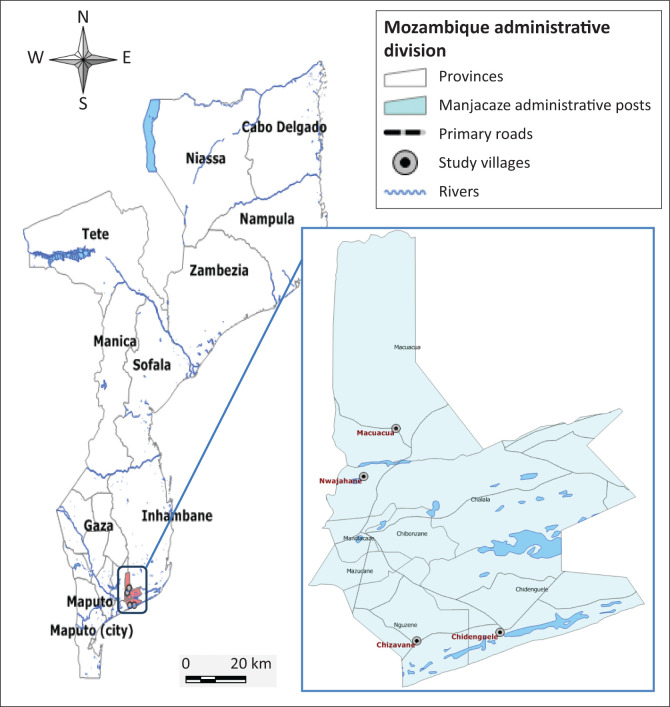
Map of Mozambique indicating the location of the study site.

From June 2020 to July 2020, a cross-sectional study was conducted in four villages of Manjakazi district, Southern Mozambique (Figure 1). The study villages were selected because public and private chicken development projects are being implemented. The farmer households were selected based on owners’ willingness to participate in the study. The required minimum sample size was calculated using the formula:


$$n=(\text{Z}\alpha^{2}\times \text{p}\times \text{q}/{\text{L}}^{2})$$ [Eqn 1]

where *n* = sample size required; Zα = 1.96 is the value required for confidence of 95%; *p* = a priori estimate of the prevalence; *q* = 1 − *p* the complementary of prior estimate; and L = 5%, the precision of estimate (Emikpe et al. 2003). A previous estimate of the prevalence of 50% was used, as there were no previous studies regarding IB and ARV in Mozambique. A total of 467 serum samples were screened to determine the seroprevalence of IBV and ARV. Blood samples were collected aseptically from the wing vein of each chicken. About 2 mL – 4 mL of blood samples were collected using a sterile disposable syringe with 22 gauge 1 and a quarter needle size. Serum was acquired after 5 min centrifugation at 1500 rotation per minute (rpm) of the coagulated blood samples and stored at −20 °C before testing.

### Serology

Serum samples were analysed using commercial enzyme-linked immunosorbent assay (ELISA) kits for the presence of anti-IBV antibodies (ProFLOK^®^ Infectious Bronchitis Virus Antibody Test Kit, Synbiotics Corp., San Diego, CA, United States) and anti-ARV antibodies (ProFLOK^®^ Avian Reovirus Virus Antibody Test Kit, Synbiotics Corp., San Diego, CA, United States) according to the manufacturer’s instructions. Optical reading was performed at 450 nm, and the cut-off values used were according to the manufacturer’s instructions.

### Statistical analysis

Data were entered into a Microsoft Excel^®^ spreadsheet (Microsoft Corporation, Redmond, Washington, United States) and exported to Stata^®^ version 12.1 (Stata IC 12.1 for Windows) software (StataCorp LLC, College Station, Texas, United States) for analysis. Prevalence data were analysed using the chi-square test (χ^2^-test). Logistic regression models were used to compute odds ratios (OR) to identify the risk for being infected as dichotomous dependent variable and independent variable (location). In all chi-square tests, *p* < 0.05 was considered statistically significant.

### Ethical considerations

Ethical review and approval were granted by the Scientific Board of the Faculty of Veterinary Medicine, Eduardo Mondlane University (ethical clearance number 280/FAVET).

## Results

Antibodies to IBV and ARV were detected in chickens from all villages included in the study. Serological results are presented in Table 1. The seroprevalences for IBV showed wide variation between villages. Chidenguele and Macuacua villages showed the highest and lowest seroprevalence, respectively. The risk of becoming exposed to IBV was significantly higher in Macuacua and Chizavane villages than Chidenguele village (*p* < 0.05). Narrowed seroprevalence variations between villages were seen for ARV. Seroprevalences of ARV did not exhibit significant differences between villages (*p* = 0.05).

**TABLE 1:** Seroprevalence of infectious bronchitis virus and avian reovirus in unvaccinated backyard chickens in Southern Mozambique.

Village	Seroprevalence				
	*n*	IBV		ARV	
		%	Exact 95% CI	%	Exact 95% CI
Nwadjahane	94	86.2	77.5–92.4	94.7	88.0–98.2
Macuacua	113	93.8	87.6–97.4	94.8	88.8–98.0
Chizavane	130	93.1	87.2–96.8	96.9	92.3–99.2
Chidenguele	130	84.6	77.2–90.3	96.2	91.2–98.7

**Total**	**467**	**89.5**	**77.2–97.4**	**95.7**	**88.0–99.2**

IBV, infectious bronchitis virus; ARV, avian reovirus; CI, confidence interval.

## Discussion

This study is the first cross-sectional survey conducted to determine the seroprevalence of IBV and ARV among backyard chickens at the village level in Southern Mozambique. In unvaccinated flocks, positive serological results are clear evidence that the birds have been exposed to the infectious agent under consideration, although without identifying the infectious strains (Chaka et al. 2012). In the present study area and elsewhere in Mozambique, backyard chickens are not vaccinated against IBV or ARV. Therefore, the presence of antibodies to IBV and ARV was considered evidence of exposure to natural infection.

This study’s findings indicated that IBV is widespread among backyard chickens in the studied villages, with a seroprevalence of 93.3%. This high prevalence agrees with reports from Zimbabwe (86%) (Kelly et al. 1994), Switzerland (> 75%) (Keller-Berger & Hoop 1993), Bangladesh (74%) (Biswas et al. 2009), central Ethiopia (91% and 97.46%) (Habte et al. 2022; Shiferaw et al. 2022) and Nigeria (82.95%) (Ijoma et al. 2022). Relatively lower IBV seroprevalences had been recorded in Mexico (56.5%) (Gutierrez-Ruiz et al. 2000), South Africa (43%) (Thekisoe et al. 2003), Botswana (65.22%) (Mushi et al. 2006) and north-west Ethiopia (23.96%) (Birhan et al. 2021). The high proportion of serologically positive samples with the absence of any clinical signs or significant mortality may imply that village chickens are resistant or had been infected by less virulent strains, as has been described in Kenya (Mbuthia et al. 2008).

To date, very few studies on seroprevalence of ARV in backyard chickens have been conducted worldwide. The present study’s findings showed that the seroprevalence of ARV is very high (94.4%), which agrees with reports from western Turkey (86.53%) (Erol & Sengül 2012) and Korea (100%) (Jae-Kyo et al. 2019). Lower ARV seroprevalences were reported from Zimbabwe (3%) (Kelly et al. 1994). Although a high seroprevalence (94.4%) of ARV was found in the present study, the importance of ARV in backyard chickens remains still to be determined.

In backyard flocks from the present study sites, biosecurity measures are not employed, flocks are often composed of a mixture of ages and species and new chickens are continually added. The authors speculate that these husbandry practices and the backyard chickens–wild birds contact may explain the high seroprevalence reported in this study for both IBV and ARV (Wille & Holmes 2020).

In summary, it is concluded that IBV and ARV are circulating among indigenous chickens in the investigated area. Therefore, the infected chickens may represent a threat in the transmission of those viruses to wild birds, broiler or layer chickens in that region. However, additional research is warranted to identify the circulating strains and the epidemiology of these diseases.
